# Prevalence of Gallstones in Ulcerative Colitis and Crohn’s Disease: A Systematic Review and Meta-Analysis

**DOI:** 10.7759/cureus.26121

**Published:** 2022-06-20

**Authors:** Mirza M. A Baig, Shayan A Irfan, Anusha Sumbal, Ramish Sumbal, Sanjay Kumar, Junaid Ahmad, Nikhila Gandrakota, Laila Tul Qadar, Maida S Chaudhry, Azka Feroz, Muhammad Sheharyar Warraich

**Affiliations:** 1 Internal Medicine, Dow University of Health Sciences, Karachi, PAK; 2 Internal Medicine, Bahria University Medical and Dental College, Karachi, PAK; 3 Internal Medicine, Liaquat University of Medical and Health Sciences, Jamshoro, PAK; 4 Family Medicine, Emory University School of Medicine, Atlanta, USA; 5 Internal Medicine, DHR Health Institute for Research and Development, Edinburg, USA; 6 Internal Medicine, John H. Stroger, Jr. Hospital of Cook County, Chicago, USA

**Keywords:** gallstones, ulcerative colitits, crohn's disease, inflmammtory bowel disease, prevalence

## Abstract

The meta-analysis aimed to investigate the prevalence of gallstones (GS) in Inflammatory bowel disease (IBD), especially ulcerative colitis (UC). A systematic and thorough search was conducted on online electronic databases (PubMed/Medline, Cochrane Library, and Google Scholar) from the databases' inception to April 30th, 2022. Review Manager 5.4.1 (The Nordic Cochrane Centre, The Cochrane Collaboration, Copenhagen) was used for all statistical analyses and forest plots. Only studies that met inclusion criteria were selected. The selected studies were pooled using a random-effect model and the results were reported in the odds ratio (OR) with their corresponding 95% confidence interval (CI). Ten studies met the final inclusion criteria and were analyzed. Patients with UC had significantly higher prevalence of GS than those in the control group (OR=1.67 [1.32-2.11]; p < 0.0001; I^2^=58%). There was also significant prevalence of GS in Crohn’s disease (CD) than those in control group (OR=2.22 [1.82, 2.69]; p < 0.00001; I^2^=31%). Analysis also showed the prevalence of GS in studies conducted in Asia (OR=2.00 [1.48, 2.70]; p < 0.00001; I^2^=80%) and Europe (OR= 1.84 [1.32, 2.55]; p = 0.0003; I^2^=45%) compared to the control group. This study provided a conclusive answer to whether GS is significant in UC or not. Our meta-analysis provides a well-powered estimate that there is a prevalence of GS in UC. CD is also significantly associated with GS.

## Introduction and background

Inflammatory bowel disease (IBD) has been a global healthcare problem [1]. Studies estimate that 2.5-3 million people suffer from IBD in Europe [2]. The health economic burden and permanent work disability due to IBD is high in Europe, with a total yearly direct healthcare cost of 4.6-5.6 billion euros [3]. IBD is a hypernym of Crohn’s Disease (CD) and ulcerative colitis (UC), which are distinct chronic bowel-relapsing inflammatory disorders [4]. UC affects the superficial mucosa, starting with the rectum, in a continuous pattern and is limited to the colon. CD is characterized by transmural inflammation that can affect any part of the GI tract from mouth to anus [2]. IBD has been associated with several extra-intestinal manifestations seen in 25% to 40% of patients with IBD patients, including peripheral arthritis, erythema nodosum, and episcleritis [5]. Those involving the extrahepatic biliary tract include gallstone disease [6].

The relationship between GS and CD has been well recognized since the 1960s, and this prevalence of CD has been estimated to be 13-14%, as reported in different series [7-10] but this same relation is subjected to variability when assessing UC. A meta-analysis by Zhang et al. clearly established a relationship between GS and CD but showed no significant association with UC [11]. Since this meta-analysis, a number of other observational studies have been published that presented varying associations of GS between UC and CD. Therefore, we aim to pool all the published data assessing the prevalence of gallstones in UC and CD separately to remove existing discrepancies amongst studies.

In the previous meta-analysis [11], studies included were from areas located in Europe. However, the epidemiology of this disease in Westernized nations is changing throughout the world at the turn of the 21st century [12]. Now, newer epidemiological studies suggest that incidence might be rising rapidly in South America, Eastern Europe, Asia, and Africa [13]. Any interruption in excretion and reabsorption of bile acids from the gut can result in the precipitation of gallstones [14-16]. Thus, an updated meta-analysis is conducted, which includes studies from other parts of the world, including Asia. The previous meta-analysis was limited by the fact that it included studies with smaller samples [11]. So we aimed to conduct an updated meta-analysis with recent studies having a much larger sample size for better, robust, and more reliable results.

The primary objective of this updated meta-analysis is to find out the prevalence of GS in patients with CD and UC. The secondary objective is to investigate if there is any geographical significance in the association of GS.

## Review

Materials and Methods

Search Strategy and Databases

Preferred Reporting Items for Systematic Review and Meta-analyses (PRISMA) guidelines and protocols were followed for conducting this meta-analysis [17]. An online electronic search from databases namely, PubMed/Medline, Cochrane Library, and Google Scholar was conducted from the inception of databases to April 30th, 2022 with only English language-based literature. In addition, studies that were cited by previous meta-analyses, cohort studies, and review articles were screened as well to identify any relevant studies. A detailed literature search is provided in Table 1.

**Table 1:** Details of the search strategy

Search Engine	Search Strategy
Pubmed/Medline	(("crohn*"[All Fields] AND ("disease"[MeSH Terms] OR "disease"[All Fields] OR "diseases"[All Fields] OR "disease s"[All Fields] OR "diseased"[All Fields])) OR "IBD"[All Fields] OR ("inflamm"[All Fields] AND ("bowel s"[All Fields] OR "bowell"[All Fields] OR "intestines"[MeSH Terms] OR "intestines"[All Fields] OR "bowel"[All Fields] OR "bowels"[All Fields]))) AND ("gallstones"[MeSH Terms] OR "gallstones"[All Fields] OR ("gall"[All Fields] AND "stones"[All Fields]) OR "gall stones"[All Fields] OR "bilestone"[All Fields] OR ("gallstones"[MeSH Terms] OR "gallstones"[All Fields] OR ("biliary"[All Fields] AND "calculus"[All Fields]) OR "biliary calculus"[All Fields]) OR ("urinary bladder calculi"[MeSH Terms] OR ("urinary"[All Fields] AND "bladder"[All Fields] AND "calculi"[All Fields]) OR "urinary bladder calculi"[All Fields] OR "cystolith"[All Fields] OR "cystoliths"[All Fields]) OR ("calculi"[MeSH Terms] OR "calculi"[All Fields] OR "concretion"[All Fields] OR "concretions"[All Fields]) OR ("cholelithiasis"[MeSH Terms] OR "cholelithiasis"[All Fields] OR "cholelithiases"[All Fields]) OR ("extra-intestinal"[All Fields] AND ("manifest"[All Fields] OR "manifestating"[All Fields] OR "manifestation"[All Fields] OR "manifestations"[All Fields] OR "manifested"[All Fields] OR "manifesting"[All Fields] OR "manifestion"[All Fields] OR "manifestions"[All Fields] OR "manifests"[All Fields])))
Cochrane	(Crohn* disease OR IBD OR inflamm * bowel) AND (gall stones OR bilestone OR biliary calculus OR cystolith OR concretion OR cholelithiasis OR extra-intestinal manifestation)
Google Scholar	(Crohn* disease OR IBD OR inflamm * bowel) AND (gall stones OR bilestone OR biliary calculus OR cystolith OR concretion OR cholelithiasis OR extra-intestinal manifestation)

Study Selection

All studies were included if they met the following eligibility criteria: (a) articles describing patients of UC, CD, or both; (b) GS should be present as an only or one of the variables that were being assessed; (c) No previous history of GS or ileal surgery should be present in the experimental or control group; (d) articles should have a defined number of patients and control.

Furthermore, the strategy for research can be given as PECOS: (1) P (Population): Inflammatory patients; (2) E (Exposure): Gallstones; (3) C (Control): cancer patients without GS; (4) O (Outcome): Prevalence of GS in IBD patients; (5) S (Studies): randomized controlled trials, cross-sectional and cohort studies published in English only.

Quality Assessment and Data Extraction From Selected Studies

Two reviewers independently performed a literature search from electronic databases and a third author was consulted to resolve any discrepancies. References of the papers were exported to the EndNote Reference Library v.X7 (Clarivate Analytics, London) and duplicates were identified and removed. 

Two separate reviewers independently extracted data and assessed the quality of included studies. Newcastle-Ottawa Scale (NOS) was used to assess the quality of the selected studies. A score >6 was considered a low bias and a score 6 or less was deemed as a significant bias.

Statistical Analysis

All statistical and analytical tests were performed using Review Manager v. 5.4.1 (The Nordic Cochrane Centre, The Cochrane Collaboration, Copenhagen). All the extracted data from selected studies were pooled using random-effects model. Analyses of results were done by calculating the odds ratio (OR) with corresponding 95% confidence intervals (CI). Leave-one-out sensitivity analysis was done to see if any study had a significant effect on overall results. As per the Cochrane handbook, the value of heterogeneity I^2^ = 25-60% was considered as moderate; 50-90% as substantial; and 75-100% as considerably high heterogeneity, and p <0.1 indicated significant heterogeneity [18]. A p-value of less than 0.05 was considered significant for all analyses. The chi-square test was used to assess any differences between the subgroups.

Results

Literature Search Results

**Figure 1:**
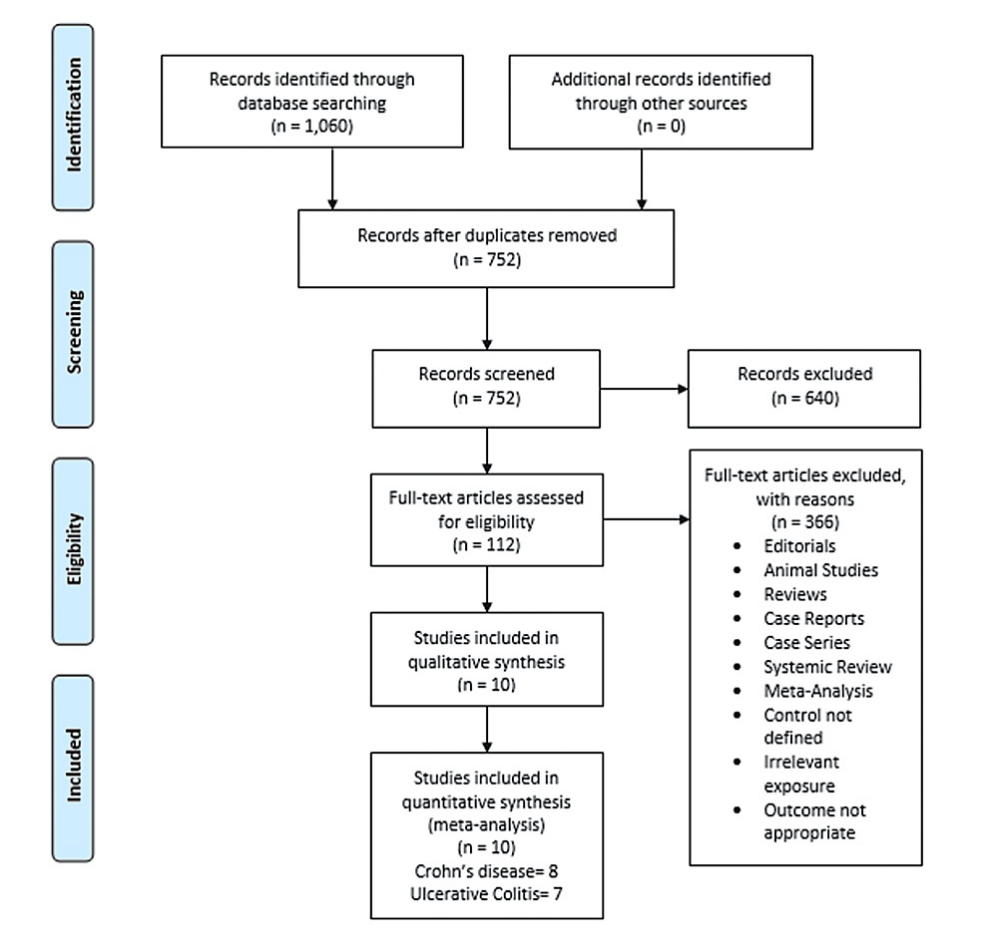
Summary of the study selection process according to PRISMA guidelines PRISMA: Preferred Reporting Items for Systematic Review and Meta-Analyses

The initial literature search from the electronic online databases brought up 1,060 potential research studies. After removal of duplicates and exclusions based on titles and abstracts, the full text of 112 studies was read for possible inclusion. A total of 10 studies remained for quantitative analysis. The summary and results of literature search are given in Figure 1.

Study Characteristics

Table 2 provides the basic characteristics of included studies. Ten studies included a total of 1,193,902 patients. The IBD group included 53,542 patients, out of which 1,155 (2.16%) had GS. The control group comprised 1,140,360 patients, and the frequency of GS in control was 8,921 (0.78%). Two separate analyses for UC and CD were conducted. The UC group included 37,567 patients, out of which 693 (1.84%) had GS. Their control group comprised 1,139,113 patients, out of which 8,588 (0.75%) consisted of GS. The CD group included 15,975 patients, out of which 462 (2.89%) had GS. The control group comprised 1,139,292 patients, out of which 8,773 (0.77%) were positive for GS.

**Table 2:** Basic characteristics of the selected studies UC, ulcerative colitis; CD, Crohn's disease; IBD, inflammatory bowel disease

Study Name	Year	Study design	Country	Basis of Diagnosis	Mean Age (years)	Female IBD patients (%)	No. IBD Patients (n)	No. Controls (Non-IBD) (n)	Odds Ratio (95% CI)	P value	NOS score
Whorwell et al. [9]	1984	Case-control	United Kingdom	Medical records	53	63.16	38	38	6.07 (1.56-23.55)	0.0092	7
Lorusso et al. [8]	1990	Case-control	Italy	Medical records	41 (UC) 39 (CD)	40.3	159	2453	1.11 (0.66- 1.87)	0.6967	9
Lapidus et al. [7]	1999	Cohort	Sweden	Medical records	N/A*	57.3	131	556	2.42 (1.55-3.76)	0.0001	8
Bargiggia et al. [19]	2003	Cohort	Italy	Medical records	38 (CD) 39 (UC)	48.5	511	145	2.25 (1.05-4.82)	0.0379	9
Parente et al. [20]	2007	Case-control	Italy	Medical records	34.7 (CD) 38.7 (UC)	45.5	600	600	1.80 (1.14- 2.84)	0.0110	9
Ha et al. [21]	2015	Cohort	Republic of Korea	Medical records	42.66	50.6	87	261	5.06 (1.99, 12.84)	0.0006	9
Jeong et al. [22]	2017	Cohort	Republic of Korea	Medical records	47.7	43.4	311	622	2.18 (1.22-3.88)	0.0083	9
Chen et al. [23]	2018	Cohort	Taiwan	ICD-9 codes	47.7	52.6	8186	8186	1.47 (1.22-1.78)	0.0001	9
Yang et al. [24]	2018	Cross-sectional	Republic of Korea	ICD-10 codes	34 (CD) 45.7 (UC)	36.5(CD) 41.9(UC)	43,281	1,127,261	2.08 (1.92-2.26)	0.0000	9
Sturdik et al. [25]	2019	Case-control	Slovak Republic	Medical records	40	47.9	238	238	1.42 (0.79-2.53)	0.2414	8

Publication Bias 

**Figure 2:**
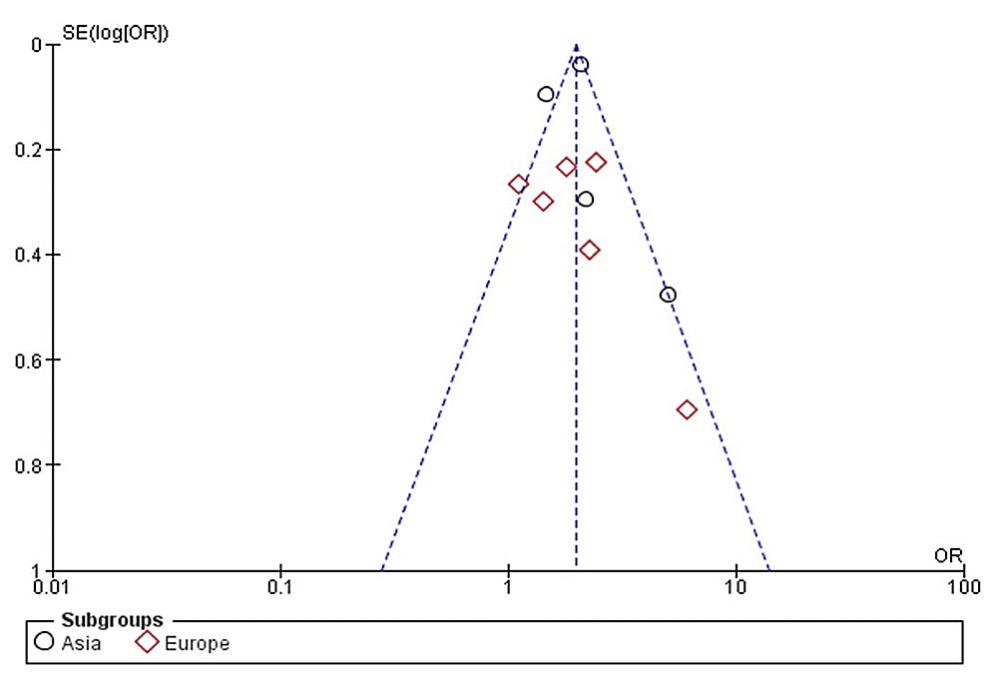
Funnel plot for publication bias

No publication bias was noted in our meta-analysis on inspection of the funnel plot as shown in Figure 2.

Quality Assessment of Included Studies

Quality assessment for observational studies was done by Newcastle-Ottawa Scale. All the studies were of high quality and had a low risk of bias. Detailed assessment with individual components is shown in Table 3.

**Table 3:** Details of Newcastle-Ottawa Scale for observational studies

Studies	Selection (Maximum 4)				Comparability (Maximum 2)	Outcome (Maximum 3)			Total score
	Representativeness of the Exposed Cohort	Selection of the Non-Exposed Cohort	Ascertainment of Exposure	Demonstration That Outcome of Interest Was Not Present at Start of Study	Comparability of Cohorts on the Basis of the Design or Analysis	Assessment of Outcome	Was Follow-Up Long Enough for Outcomes to Occur	Adequacy of Follow Up of Cohorts	
Lapidus et al. [7]	1	1	1	1	1	1	1	1	8
Bargiggia et al. [19]	1	1	1	1	2	1	1	1	9
Ha et al. [21]	1	1	1	1	2	1	1	1	9
Jeong et al. [22]	1	1	1	1	2	1	1	1	9
Chen et al. [23]	1	1	1	1	2	1	1	1	9
Whorwell et al. [9]	1	1	1	1	1	1	1	1	8
Lorusso et al. [8]	1	1	1	1	2	1	1	1	9
Parente et al. [20]	1	1	1	1	2	1	1	1	9
Sturdik et al. [25]	1	1	1	1	2	1	1	1	9
Yang et al. [24]	1	1	1	1	2	1	1	1	9

Results of the Meta-Analysis

**Figure 3:**
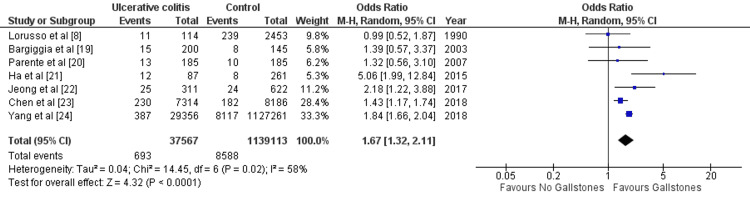
Forest plot summarizing analysis of prevalence of gallstone in patients with ulcerative colitis 95% CI, 95% confidence interval

**Figure 4:**
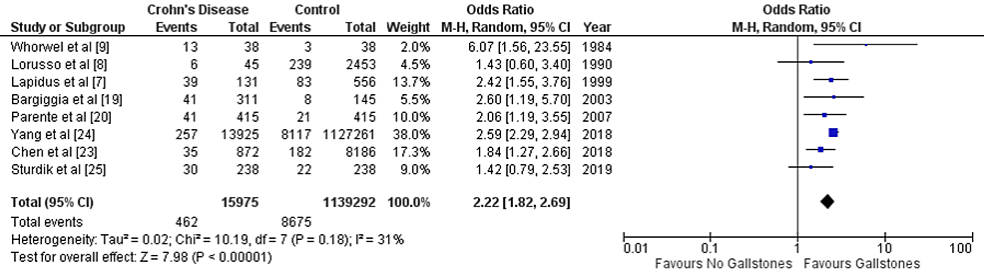
Forest plot summarizing analysis of prevalence of gallstone in patients with Crohn’s disease 95% CI, 95% confidence interval

**Figure 5:**
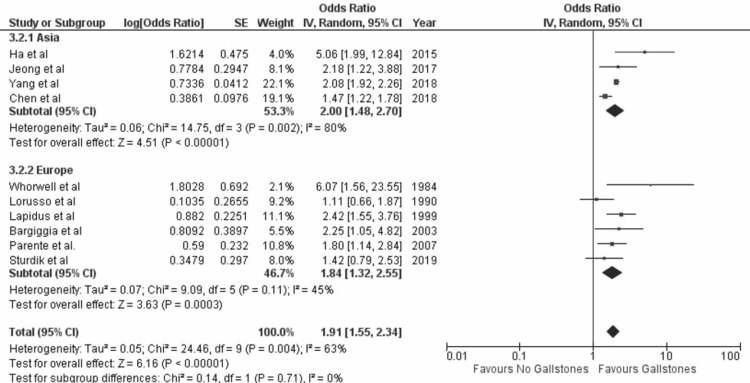
Forest plot summarizing analysis of prevalence of gallstones in Asia and Europe 95% CI, 95% confidence interval

(i) Ulcerative colitis: Seven studies reported data for the prevalence of GS in UC. Pooled result (Figure 3) showed statistically significant prevalence of GS in UC than that of in control group (OR=1.67 [1.32, 2.11]; p < 0.0001; I^2^=58%). 

(ii) Crohn’s disease: Eight studies reported data for the prevalence of GS in CD. Pooled result (Figure 4) showed statistically significant prevalence of GS in CD than that of in control group (OR= 2.22 [1.82, 2.69]; p < 0.00001; I^2^=31%).

(iii) Geographical location: Out of 10 studies, four were from Asia (three were from the Republic of Korea and one from Taiwan), and five were from Europe (three were from Italy, one from the Slovak Republic, one from Sweden, and one from the United Kingdom). Analysis (Figure 5) showed that there was higher prevalence of GS in studies conducted in Asia (OR= 2.00 [1.48, 2.70]; p < 0.00001; I^2^=80%) and in Europe (OR= 1.84 [1.32, 2.55]; p =0.0003; I^2^=45%) than the control group.

Sensitivity Analysis

A sensitivity analysis was conducted to assess the influence of each study on the overall effect by excluding one study at a time, followed by the generation of pooled OR for the rest of the studies. It is vital to note that Yang et al. had around a million population in its study but still removing it in ulcerative colitis (OR= 1.63 [1.15, 2.31]; p =0.006; I^2^=51%), Crohn’s disease (OR= 2.00 [1.62, 2.47]; p < 0.00001; I^2^=0%) and in Asia (OR= 2.20 [1.21, 4.02]; p =0.01; I^2^=74%) did not change the overall outcome which showed that the outcome was not influenced by this study [24]. On leave-one-out analysis, no significant change was seen in the p-value after removing studies one by one. This showed that the results were robust.

Discussion

This 53,542 IBD patients’ analysis shows an instrumental result regarding the prevalence of GS in IBD patients. Although a previously published meta-analysis by Zhang et al. (2015) has explored this association, their results were limited by the small sample size (1,439 IBD patients). It also failed to establish any significant association between GS and UC [11].

A very prominent finding in our analysis is the association between GS and UC. Some articles have suggested and presented a statistically significant prevalence of GS in UC [8], but no previous meta-analysis has confirmed this outcome. This analysis provides a well-established result, highlighting the prevalence of GS in CD and UC. Based on our findings, it is suggestive that IBD patients have a risk of developing GS. Physicians should provide medication and proper lifestyle modification, which will improve the quality of life of IBD patients and act as prophylaxis for GS prevention.

It is pivotal to provide reasons for GS in CD and UC. Enterohepatic circulation is responsible for the excretion of bile acids by the liver into the small intestine, and then cholesterol is excreted through bile [14]. Any interruption in the mechanism of excretion and reabsorption of bile acids from the intestine results in the precipitation of gallstones. CD disrupts the enterohepatic circulation in the terminal ileum and can slow down the gallbladder contractility; therefore, it can lead to the formation of gallstones [15,16].

Several controversial studies show different results regarding the prevalence of GS in UC; however, a study conducted by Holmquist et al. showed that an affected ascending colon in UC could increase fecal bile excretion; therefore, loss of excess bile will result in precipitation of GS [26]. Several studies also showed gallstone development following the colectomy for UC [27], suggesting that the colon plays a minor role in bile reabsorption. However, the mechanism in the development of GS in UC is still disputed.

The studies showed the influence of the geographical variations of GS prevalence in IBD, highlighting the increasing incidence of GD in the Asia and Europe region [12-13]. Although no large-scale and diverse studies have been conducted that can profoundly state regional influence on GS. Our subgroup analysis shows that there is statistical significance in regions of Asia and Europe for the presence of GS.

Based on our findings, it is suggestive that IBD patients have a risk of developing GS, which ultimately leads to complications such as choledocholithiasis, acute cholangitis, and gallstone ileus, which in some cases can be life-threatening as it may proceed to acute biliary pancreatitis and gallbladder carcinoma [28]. This research will help physicians to better manage IBD patients for future occurrence of GS by an annual screening of the gallbladder via ultrasound. Also, physicians can prophylactically administer IBD patients with lipid-lowering agents such as statin drugs, along with a restricted cholesterol diet. A selected subgroup of patients with asymptomatic gallstones but who are at high risk of developing symptoms of gallbladder cancer or biliary pancreatitis can also be managed by prophylactic cholecystectomy [28].

Our study is limited by some factors such as (a) all studies were observational in nature, the results of which can have some bias (b) controls selected by some studies were based on hospital settings which might have overestimated gallstone formation. Further research is needed especially with more randomized studies.

## Conclusions

This study provides a conclusive answer to whether GS is significant in UC or not. Our meta-analysis provides a well-powered estimate that there is a prevalence of GS in UC. CD is also significantly associated with GS. Although patients of CD and UC have overlapping clinical symptoms, patients with predominant UC symptoms also have a possibility of gallstones and should be kept in mind when presenting with right upper quadrant pain and other symptoms of cholelithiasis.
